# Impact of Obesity on Mortality in Adult Trauma Patients

**DOI:** 10.7759/cureus.13352

**Published:** 2021-02-15

**Authors:** Blake Drury, Christopher Kocharians, Fanglong Dong, Louis Tran, Shawhin Beroukhim, Reza Hajjafar, Richard Vara, David Wong, Brandon Woodward, Michael M Neeki

**Affiliations:** 1 Emergency Medicine, Arrowhead Regional Medical Center, Colton, USA; 2 Medicine, Western University of Health Sciences, Pomona, USA; 3 Surgery, Arrowhead Regional Medical Center, Colton, USA; 4 Surgery, California University of Science and Medicine, San Bernardino, USA; 5 Emergency Medicine, California University of Science and Medicine, San Bernardino, USA

**Keywords:** body mass index: bmi, blunt truma, penetrating trauma, mortality

## Abstract

Introduction

Trauma is a major cause of morbidity and mortality amongst all populations in the United States. With the widespread increase of obesity in the United States, studies have been conducted to compare different body mass index (BMI) groups and their clinical outcomes for traumatic injuries. The goal of this study was to retrospectively compare mortality between adult trauma patients with a high BMI to those with a lower BMI as well as investigate whether the mechanism of trauma had an effect on the outcome.

Methods

This study was a retrospective review of all adult trauma patients presented to the emergency department at Arrowhead Regional Medical Center (ARMC) between January 2014 and October 2019. The outcome was all-cause mortality. Patients were grouped according to BMI and mechanisms of injury, including blunt trauma, low velocity penetrating trauma, and high velocity penetrating trauma. Patients were also stratified by injury severity scores (ISS).

Results

Among the 9642 patients assessed in this study, majority (88%) of patients sustained blunt trauma. The number of patients among the three different BMI groups was appropriately equal with 34.4% of normal BMI, 34.6% overweight, and 31.1% obese. The overall mortality of all patients studied was 2.6% (n=248). There was no statistically significant difference in mortality among the three different BMI groups for blunt trauma, penetrating trauma, and subgroup analyses stratified by ISS score (ISS<16 or ISS ≥ 16).

Conclusion

Our study found no statistically significant differences in mortality among the three BMI groups in regard to mortality, even when stratified by ISS, or mechanism of injury, and traumatic velocities.

## Introduction

Trauma is a leading cause of death and disability in the United States (US), ranking only behind heart disease and cancer [1]. In 2017, the Centers for Disease Control and Prevention reported that deaths from unintentional injuries was nearly 170,000 [2]. The economic burden of trauma-related injuries is also high. Accounting for both direct healthcare cost and lost productivity, the impact to the US economy has been estimated to be $671 billion annually [3].

The rising prevalence of obesity in the US is another major economic burden to the healthcare system. One study estimated that obesity accounts for 21% of all healthcare-related expenses [4]. Obesity is also a significant comorbidity among patients who suffer traumatic injuries. Obese patients have a 25% increased risk of workplace injury [5]. When compared to patients of normal weight (body mass index, BMI<18.5 kg/m^2^), the odds of sustaining an injury were found to be up to 48% higher in overweight and obese individuals [6].

The effect of obesity in trauma patients has been debated. The obesity paradox is the association of decreased mortality of obese patients in certain conditions. Some have argued that having an increased BMI may confer a protective effect in trauma [7,8]. However, others have contended that an increased BMI makes no difference or may lead to a higher mortality rate [9-11]. This study aimed to examine the difference in mortality with different BMI groups among adult trauma patients. Furthermore, this study also investigated if the mechanism and severity of injury would impact the mortality rates among the different BMI groups.

## Materials and methods

This study was approved by the Institutional Review Board at Arrowhead Regional Medical Center (ARMC). ARMC is a 456-bed acute care teaching facility and American College of Surgeons certified level II trauma center located in San Bernardino County, California. The ARMC emergency department (ED) is one of the busiest in the state of California with more than 100,000 visits and more than 3,000 adult traumas annually. All patients with traumatic injuries were evaluated and managed by qualified providers in accordance with Advanced Trauma Life Support (ATLS) [12].

This was a retrospective chart review of patients who visited ARMC ED between January 2014 and October 2019. Inclusion criteria for this study consisted of adult trauma patients of 18 years and older who sustained penetrating or blunt trauma. Patients were excluded if they were under the age of 18, did not sustain blunt or penetrating trauma, or did not have a clear recorded BMI in the electronic medical record. The primary objective of this study was to determine the association between mortality at hospital discharge and BMI groups. Data were separated into three BMI groups: normal weight (BMI 18.5-24.9 kg/m^2^), overweight (BMI 25-29.9 kg/m^2^), and obese (BMI 30+ kg/m^2^). Additional variables assessed in this study included age, sex, injury severity score (ISS), mechanism of injury (blunt vs penetrating), and traumatic velocities (low- vs high-velocity injury). Low-velocity penetrating injuries refer to those items that produce less tissue distraction, and high-velocity penetrating injuries refer to those items that produce wider tissue distraction as a result of high level of kinetic energy, similar to those definitions used by Kuhajda et al. [13].

All statistical analyses were conducted using the SAS software for Windows version 9.4 (Cary, North Carolina, USA). Descriptive statistics were presented as means and standard deviations for continuous variables, along with frequencies and proportions for categorical variables. Chi-square tests were conducted to assess the association between the categorical outcomes and the three BMI categories. All statistical analyses were two-sided. P-value < 0.05 was considered to be statistically significant.

## Results

**Figure 1:**
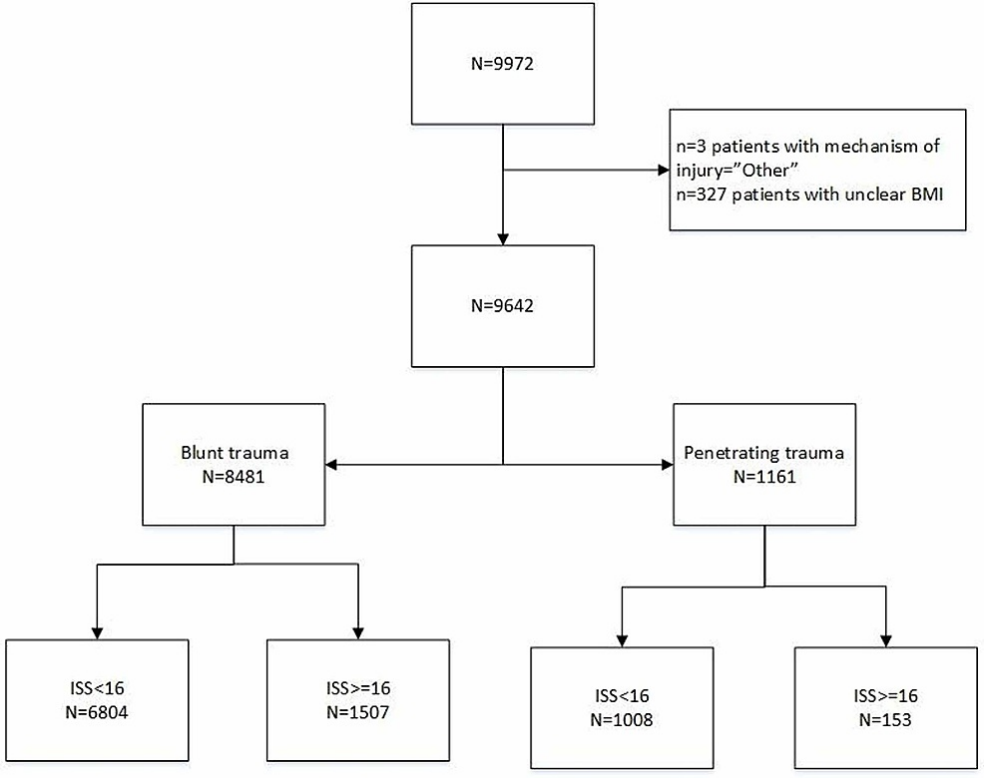
Patient flow chart ISS: injury severity scores; BMI: body mass index.

A total of 9972 patients were included in the original database. Three patients were excluded for unclear documentation on the mechanisms of injury. An additional 327 patients were excluded for insufficient information regarding their BMI. As a result, a total of 9642 patients were included in the final analysis. Figure 1 presents the detailed patient flow chart. 

Table 1 presents the descriptive summaries for these 9642 patients. The average age was 46.14 (SD=20.4) years. The average ISS score was 9.54 (SD=8.2). Majority of patients were male (70.9%, n=6837) and sustained blunt trauma (88.0%, n=8481). Proportions of each BMI category were approximately equal, with 34.4% being normal BMI, 34.6% being overweight, and 31.1% being obese. The overall mortality rate was 2.6% (n=248).

**Table 1:** Demographic summary of patients ISS: injury severity scores; BMI: body mass index.

BMI	28.31 ± 6.87	
ISS	9.54 ± 8.2	
Age	46.14 ± 20.4	
Gender	
Female	2805 (29.1%)	
Male	6837 (70.9%)	
Mechanism of Injury	
Blunt	8481 (88%)	
Penetrating	1161 (12%)	
BMI Group	
18.5-24.9 kg/m^2^	3314 (34.4%)	
25-29.9 kg/m^2^	3334 (34.6%)	
30+ kg/m^2^	2994 (31.1%)	
ISS group	
< 16	7812 (82.5%)	
≥ 16	1660 (17.5%)	
Missing ISS values	170	
Discharge Disposition	
Alive	9394 (97.4%)	
Dead	248 (2.6%)	

Analyses were conducted to assess the association between BMI and mortality in patients sustaining different mechanisms of injury. Table 2 presents the analysis results. For both blunt and penetrating trauma combined, there was no statistically significant difference in mortality between the three BMI groups (p=0.3420). There was no statistically significant difference in mortality between the three BMI groups within the ISS<16 group (p=0.2335) and within the ISS ≥ 16 group (p=0.9319).

**Table 2:** Overall mortality by mechanism of injury and ISS group *All values are presented as number of mortality/number of patients in that strata with the corresponding percentage inside the parenthesis. ISS: injury severity scores; BMI: body mass index.

		BMI 18.5-24.9	BMI 25-29.9	BMI 30+	p-value
Overall Analysis					
Both Blunt and Penetrating combined	Overall	96/3314 (2.9%)	81/3314 (2.4%)	71/3314 (2.4%)	0.3420
	ISS < 16	29/2660 (1.1%)	19/2660 (0.7%)	18/2660 (0.7%)	0.2335
	ISS ≥ 16	65/594 (10.9%)	61/594 (10.3%)	52/594 (10.9%)	0.9319
Subgroup Analysis					
Blunt Trauma	Overall	84/2901 (2.9%)	76/2900 (2.6%)	63/2680 (2.4%)	0.4457
	ISS < 16	28/2300 (1.2%)	18/2320 (0.8%)	15/2184 (0.7%)	0.127
	ISS ≥ 16	54/541 (10%)	57/528 (10.8%)	47/438 (10.7%)	0.892
Penetrating Trauma	Overall	12/413 (2.9%)	5/434 (1.2%)	8/314 (2.6%)	0.1821
high velocity	ISS < 16	0/143 (0%)	0/105 (0%)	0/103 (0%)	N/A
	ISS ≥ 16	11/41 (26.8%)	4/41 (9.8%)	5/35 (14.3%)	0.1057
low velocity	ISS < 16	1/126 (0.8%)	1/151 (0.7%)	3/102 (2.9%)	0.3235
	ISS ≥ 16	0/10 (0%)	0/14 (0%)	0/2 (0%)	N/A

Two subgroup analyses were conducted to further assess the association between BMI and mortality. The results were also presented in Table 2. For blunt trauma overall, there was no statistically significant difference in mortality between the three BMI groups (p=0.4457). Within each subgroup analysis, there was no statistically significant difference in mortality between the three BMI groups (p=0.127 for blunt trauma with ISS<16 subgroup, p=0.892 for blunt trauma with ISS ≥ 16).

For all penetrating traumas, there was no statistically significant difference in mortality between the three BMI groups (p=0.1821). The overweight BMI group had the lowest mortality rate (1.2%); whereas, the normal BMI group had the highest mortality rate (2.9%) overall. For each subgroup analysis, there was no statistically significant difference in mortality between the three BMI groups (all p-values>0.05).

## Discussion

There has been inconsistent findings regarding the effect of obesity on mortality among trauma patients. One study observed that patients with higher BMI had a lower mortality in injuries from falls, but also had longer hospital length of stay and a decreased likelihood to be discharged home [7]. Another study examined patients with blunt abdominal trauma and found that morbidly obese patients (BMI ≥ 40 kg/m^2^) are less likely to have a gastrointestinal injury or need an operative intervention for hollow viscus injury when compared with underweight patients [14]. Treto et al. reported that mortality after blunt trauma was significantly higher in patients classified as underweight (BMI < 18.5 kg/m^2^) when compared to those with normal BMI [15]. Similarly, Bloom and colleagues found that patients with higher BMI are less likely to have severe injuries or need an operation after sustaining an abdominal stab wound. They noted that patients with BMI < 18.5 kg/m^2^ required an operation three times more often than those with BMI > 35 kg/m^2^ [8].

Various theories on the protective effect of obesity in trauma have been proposed. One hypothesis is that the extra subcutaneous fat seen in obese patients functions similarly to an airbag, absorbing the forces generated in a collision. Additionally, the extra adipose tissue in obese patients provides a greater distance to the internal organs, which may be protective in penetrating injuries [16]. Patients who are morbidly obese therefore have a mechanical and physical protective barrier for both blunt and penetrating traumas. Our study leans toward supporting this theory. While not statistically significant, the highest mortality rates in our study were found in the groups with the lowest BMI.

In contrast, other investigators came to the opposite conclusion in their research outcomes. Neville and colleagues noted a higher incidence of multiple organ failure and mortality rate among obese patients admitted to the intensive care unit following blunt trauma [17]. Additional investigators also reported that obesity is associated with prolonged duration of mechanical ventilation, ICU, and hospital length of stay [18]. Other researchers have suggested that the proinflammatory state, metabolic syndrome, and cardiopulmonary pathologies in obesity may lead to increased complications and mortality following trauma [19]. Obese patients have decreased pulmonary function due to changes in lung volume, compliance, and ventilation/perfusion mismatches [20].

Obesity is also an independent risk factor for cardiovascular disease. It is associated with a higher incidence of insulin resistance and deep vein thrombosis [6,21]. One study of more than 46,000 patients noted that respiratory, thromboembolic, and infectious complications were more common in obese trauma patients. The study concluded that obesity is associated with increased mortality following trauma [9]. In contrast, our retrospective study found no significant difference among various BMI groups with regards to overall mortality due to traumatic injuries. These findings held true when stratified for the mechanism of injury, including blunt vs penetrating injuries. Duane and colleagues reported that mortality rates were not found to be different between obese versus non-obese patients after blunt trauma [22]. Nash et al. reported no statistically significant difference between obese and non-obese patients when comparing operative approach, injury location, and postoperative outcomes for penetrating trauma [16]. While obese patients do have increased infectious morbidity, wound dehiscence, and a prolonged length of stay, increased BMI was not found to be an independent predictor of increased morbidity or mortality after trauma laparotomy [23].

Limitations

Trauma is a complex and diverse disease with a wide range of presentations. It is nearly impossible to properly compare the severity of sustained injuries in a retrospective study using the ISS. The ISS has been criticized for underestimating the severity of the trauma and can cause statistically significant differences in mortality with the same score generated [24,25]. A severely injured brain is not managed the same way as a severely injured lung, liver, or bowel. Furthermore, the impact of obesity will vary depending on the type and anatomical location of the injury. While the excess adipose tissue may confer some protection for abdominal trauma, it may do nothing for head trauma.

Similarly, obesity is a complex disease and can have diverse physiologic and anatomic complications. Since obesity predisposes patients to many other comorbidities, these may need to be scrutinized individually. Specific areas for investigations may need to take into account the presence of pre-existing conditions as well as the development of complications related to obesity during hospital stay. 

## Conclusions

The study found no statistically significant difference in mortality among the three BMI groups, even when stratified by blunt versus penetrating traumas, ISS, and traumatic velocities. Evidence suggests that there is a multifactorial process that occurs amongst obese patients with traumatic injuries. Further prospective studies are needed to clarify and evaluate the specifics of the pathophysiological process after a traumatic event in a different BMI population.
